# Characterization of human peritoneal monocyte/macrophage subsets in homeostasis: Phenotype, GATA6, phagocytic/oxidative activities and cytokines expression

**DOI:** 10.1038/s41598-018-30787-x

**Published:** 2018-08-24

**Authors:** Antonio José Ruiz-Alcaraz, Violeta Carmona-Martínez, María Tristán-Manzano, Francisco Machado-Linde, María Luisa Sánchez-Ferrer, Pilar García-Peñarrubia, María Martínez-Esparza

**Affiliations:** 1 Departamento de Bioquímica, Biología Molecular (B) e Inmunología. Facultad de Medicina, IMIB and Regional Campus of International Excellence “Campus Mare Nostrum”, Universidad de Murcia, Murcia, Spain; 2 Servicio de Ginecología y Obstetricia, Hospital Clínico Universitario Virgen de la Arrixaca, IMIB, Murcia, Spain

## Abstract

Peritoneal macrophages play a critical role in the control of infectious and inflammatory diseases. Although recent progress on murine peritoneal macrophages has revealed multiple aspects on their origin and mechanisms involved in their maintenance in this compartment, little is known on the characteristics of human peritoneal macrophages in homeostasis. Here, we have studied by flow cytometry several features of human peritoneal macrophages obtained from the peritoneal cavity of healthy women. Three peritoneal monocyte/macrophage subsets were established on the basis of CD14/CD16 expression (CD14^++^CD16^−^, CD14^++^CD16^+^ and CD14^high^CD16^high^), and analysis of CD11b, CD11c, CD40, CD62L, CD64, CD80, CD86, CD116, CD119, CD206, HLA-DR and Slan was carried out in each subpopulation. Intracellular expression of GATA6 and cytokines (pro-inflammatory IL-6 and TNF-α, anti-inflammatory IL-10) as well as their phagocytic/oxidative activities were also analyzed, in an attempt to identify genuine resident peritoneal macrophages. Results showed that human peritoneal macrophages are heterogeneous regarding their phenotype, cell complexity and functional abilities. A direct relationship of CD14/CD16 expression, intracellular content of GATA6, and activation/maturation markers like CD206 and HLA-DR, support that the CD14^high^CD16^high^ subset represents the mature phenotype of steady-state human resident peritoneal macrophages. Furthermore, increased expression of CD14/CD16 is also related to the phagocytic activity.

## Introduction

Macrophages are a versatile and heterogeneous cell population that interconnects innate and adaptive immunity and plays a crucial role in many inflammatory diseases, tissue remodelling and wound healing, among others. These cells reside in all tissues in homeostasis, and are also rapidly recruited and differentiated from circulating blood monocytes in response to local danger signals provided by inflammation or microbial invasion^1,2^. Peritoneal macrophages are key players in the control of infectious and inflammatory diseases^3^ through a variety of effector and regulatory functions^4^. Currently, the majority of studies on phenotypic and functional characteristics of tissue resident macrophages have been performed in murine peritoneal macrophages (pMφ)^5^ or *in vitro* differentiated human blood monocyte-derived macrophages^6^. However, the population of blood monocytes from healthy people contains three subpopulations displaying different phenotypes and functions^7^ named as “classical” CD14^++^CD16^−^, “intermediate” CD14^++^CD16^+^ and “non-classical” CD14^+/low^CD16^+^ monocytes^8–10^. The CD16^+^ subpopulations are considered as pro-inflammatory monocytes since they are increased in acute^11^ and chronic^12–15^ inflammatory pathologies. These cells produce *in vitro* TNF-α, IL-6, MIP1α and MIP1β pro-inflammatory cytokines in response to LPS^7,14,16^ and they are probably expanded from the classical to the intermediate subset and from this to the non-classical subpopulation^10^. Nowadays, a great interest has been focused to uncover the specific monocyte subpopulation that gives rise to tissue resident macrophages in steady-state or in different clinical scenarios. We have recently identified a new subset of monocyte/macrophages from ascites of cirrhotic patients displaying high expression of CD14 and CD16 referred to as CD14^high^CD16^high^, bigger size and more complexity than CD16^−/low^ cells, which is not detected in peripheral blood monocytes^17^.

On the other hand, all knowledge on the origin and development of tissue resident macrophages has been obtained from experimental mouse models. In this regard, it has been recently demonstrated the existence of several types of tissue-resident macrophages independent of hematopoietic stem cell origin, which are directly derived from embryonic progenitors (yolk sac and foetal liver) and have the intrinsic capacity to proliferate and self-renew^18,19^. Furthermore, it has also been described that the expression of transcription factor GATA-binding protein 6 (GATA6) is mainly restricted to the long-lived murine F4/80^hi^CD11b^hi^ large peritoneal-resident macrophages (LPM), which retain cells of embryonic origin for at least four months, while the other subset of F4/80^low^MHC-II^hi^ referred to as small peritoneal macrophages (SPM) is derived from inflammatory monocytes^20^. Given that the new human CD14^high^CD16^high^ subpopulation of ascitic cells is not detected in peripheral blood monocytes^17^, we hypothesize that it could represent the phenotypic signature of genuine human resident-peritoneal macrophages. To assess this, we have analyzed the phenotype and the intracellular expression of GATA6 together with the phagocytic and oxidative activity, the intracellular expression of pro-inflammatory cytokines IL-6 and TNF-α, and the anti-inflammatory IL-10 in the three CD14/CD16 subsets of peritoneal monocytes/macrophages (pMo/Mφ) from healthy women. Our results confirm the presence of three CD14/CD16 subpopulations of monocytes/macrophages in steady-state. Although, the percentage of GATA6 expressing cells is similar among the three described subpopulations of pMo/Mφ, the more complex CD14^high^CD16^high^ subset contains the highest number of GATA6-expressing cells, as well as higher phagocytic/oxidative activities and expression of membrane receptors involved in antigen presentation, activation, co-stimulation and phagocytosis. The intermediate subset expresses a medium level of those markers, while the classical-like subset is more similar to the corresponding population of blood monocytes. Finally, among them a similar set of pro-inflammatory M1 and anti-inflammatory M2 polarization markers was observed, which is compatible with a basal pre-activated state to rapidly respond against any challenge to maintain peritoneal homeostasis.

## Results

### Subjects baseline characteristics

A consecutive series of 79 women was initially recruited for the study. Seven patients were excluded after diagnosis of endometriosis (n = 5) and carcinoma (n = 2). Additionally, 21 patients were excluded due to technical difficulties in the collection of peritoneal samples, such as blood contamination (n = 8) or very low amount of cell content (n = 13). Finally, 51 women fulfilling inclusion and exclusion criteria were included in the study. Clinical and analytical characteristics of blood leukocytes from people analysed are detailed in Table 1. As it can be observed, the values of analysed data correspond to the normal range for the sex and age of the group studied.

**Table 1:** Clinical and analytical characteristics of individuals included in the study.

Characteristics	Studied subjects	Healthy range
Age (Years ± SEM)	40.3 ± 11.3	
**Cell type/mm**^3^ (Mean ± SEM)		
Leukocytes	9.1 ± 2.4	4–10
Monocytes	0.73 ± 0.3	0.1–1
PMNs	5.8 ± 1.9	2–7
Platelets	239.2 ± 57.8	130–400

Data represents the Mean ± SEM of years (Age) or cell concentrations as cells/mm^3^ (Blood cell types). Normal ranges of blood cell types’ concentrations in healthy conditions are also shown as a reference.

### Human peritoneal macrophages subpopulations based on the expression of CD14 and CD16

**Figure 1:**
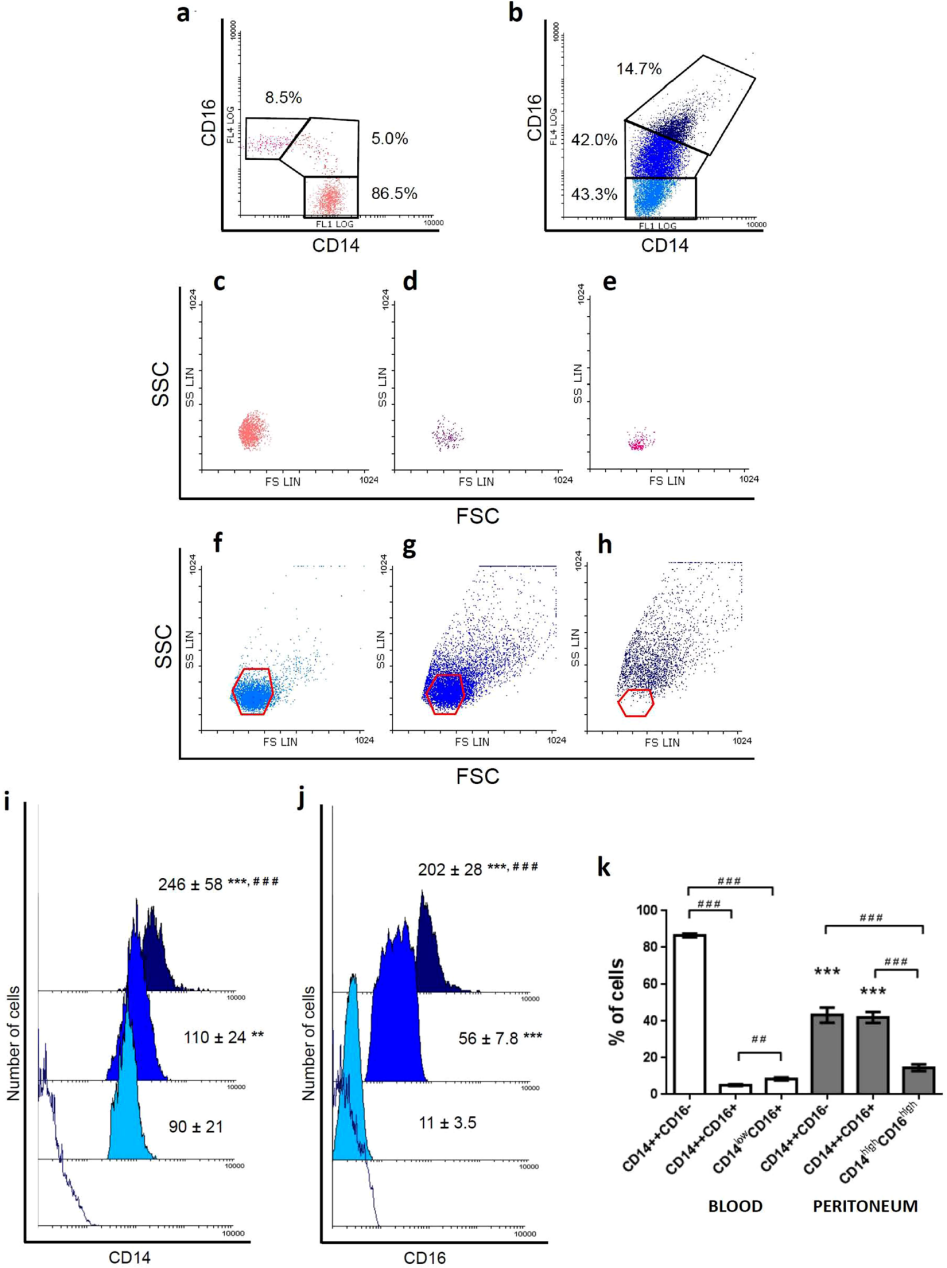
Flow Cytometry analysis of human peritoneal monocyte/macrophage subsets based on the CD14/CD16 expression pattern and Forward/Side Scattering compared to blood. Representative FACS dot-plot of CD14 and CD16 expression pattern of blood monocytes and gates selecting the three different CD14/CD16 subsets (CD14^++^CD16^−^, CD14^++^CD16^+^ and CD14^+/low^CD16^+^) is shown (**a**). Representative FACS dot-plot of CD14 and CD16 expression pattern of peritoneal monocytes/macrophages and gates selecting the three different CD14/CD16 subsets (CD14^++^CD16^−^, CD14^++^CD16^+^ and CD14^high^CD16^high^) are shown (**b**). Percentages refer to the mean of cell subpopulations gated on the total population of monocyte/macrophage cells. Comparison of the morphological distribution of blood monocytes (**c**,**d**) and peritoneal monocyte/macrophage subsets (**f**–**h**) is also displayed as dot-plots. For comparative purposes, overlay red colour polygons indicate the limits of the morphological regions in which the corresponding blood monocytes are included: CD14^++^CD16^−^ (**f**), CD14^++^CD16^+^ (**g**) and CD14^+/low^CD16^+^ (**h**). Representative FACS mean fluorescence intensity (MFI) histograms and mean MFI ± SEM of CD14 (**i**) and CD16 (**j**) expression within each peritoneal monocyte/macrophage subpopulation are displayed partially overlaid. Empty histogram shows the corresponding isotype controls, light blue histograms correspond to CD14^++^CD16^−^ cells, bright blue to the CD14^++^CD16^+^ subset, and dark blue to the CD14^high^CD16^high^ subset. Mann-Whitney U test: **p < 0.01, ***p < 0.001, between the classic-like CD16 negative subset and the two other CD16 positive subsets present in peritoneum; ^###^p < 0.001, between the two CD16 positive subsets (CD14^++^CD16^+^ *vs*. CD14^high^CD16^high^). Percentages of cell subsets were comparatively analyzed in blood (N = 29) and peritoneum (N = 36) from healthy individuals (**k**). Histograms represent mean ± SEM of every cell subset. White bars are used for blood monocyte subsets and grey bars for peritoneal monocyte/macrophage subsets. Mann-Whitney U test: ***p < 0.001, between classic and intermediate subsets present in blood *vs*. peritoneum; ^##^p < 0.01, ^###^p < 0.001, between phenotypic subsets inside each location.

First, we studied the distribution of pMo/Mφ according to the expression pattern of membrane markers CD14 (co-receptor of LPS) and CD16 (FcγRIII) in comparison to the subsets of monocytes found in blood (Fig. 1a–h). The gating strategy of blood monocytes and pMo/Mφ was carried out as follows: Initially, cells were selected according to morphology (forward *vs*. side scatter). Afterwards, cellular aggregates were excluded based on FSC-W *vs*. FSC-A. Finally, cells were gated based on CD14/CD16 expression (Supplementary Fig. S1). Results showed that, similarly to blood monocytes, pMo/Mφ are distributed into three different subpopulations in terms of CD14/CD16 expression. Results showed that differently to blood monocytes (Fig. 1a), which display the known classical CD14^++^CD16^−^, intermediate CD14^++^CD16^+^, and non-classical CD14^+/low^CD16^+^ subsets with uniform morphology and low complexity (Fig. 1c–e), pMo/Mφ, as it is displayed in Fig. 1b, are distributed into: (a) a classical-like CD14^++^CD16^−^ subset (Fig. 1f) similar to their counterparts found in blood (Fig. 1c); (b) an intermediate CD14^++^CD16^+^ subset displaying higher level of CD14 expression, and higher heterogeneity related to both granularity/complexity and size (Fig. 1g) than intermediate blood monocytes (Fig. 1d); and (c) a totally different CD14^high^CD16^high^ subset (Fig. 1h), which presents not only higher expression of both CD14 and CD16, but also includes cells with the biggest size and highest granularity.

Quantitative analysis of the MFI for CD14 (Fig. 1i) and CD16 (Fig. 1j) within each pMo/pMφ subpopulation was performed. Frequencies of the three subsets in blood *vs*. peritoneum (Mean ± SEM) are shown in Fig. 1k. As expected, the predominant subpopulation in blood was the classical subset (86.5 ± 1.0%), followed by the non-classical subset (8.5 ± 0.8%), and finally the intermediate subset with the lowest proportion (5.0 ± 0.4%); differences were significant in all cases (#). Besides, differences between each CD14^++^CD16^−^ and CD14^++^CD16^+^ subset of blood monocytes with those of pMo/Mφ were significant in both cases (*). Percentages of peritoneal classical-like and intermediate subpopulations were similar (43.3 ± 4.1% and 42.0 ± 3.0%, respectively), while the most complex subset represented a 14.7 ± 1.8% of the whole monocyte/macrophage population. Differences with percentages of the former two subpopulations were significant (#).

### Phenotypic characteristics of human peritoneal subpopulations compared to blood monocytes

**Figure 2:**
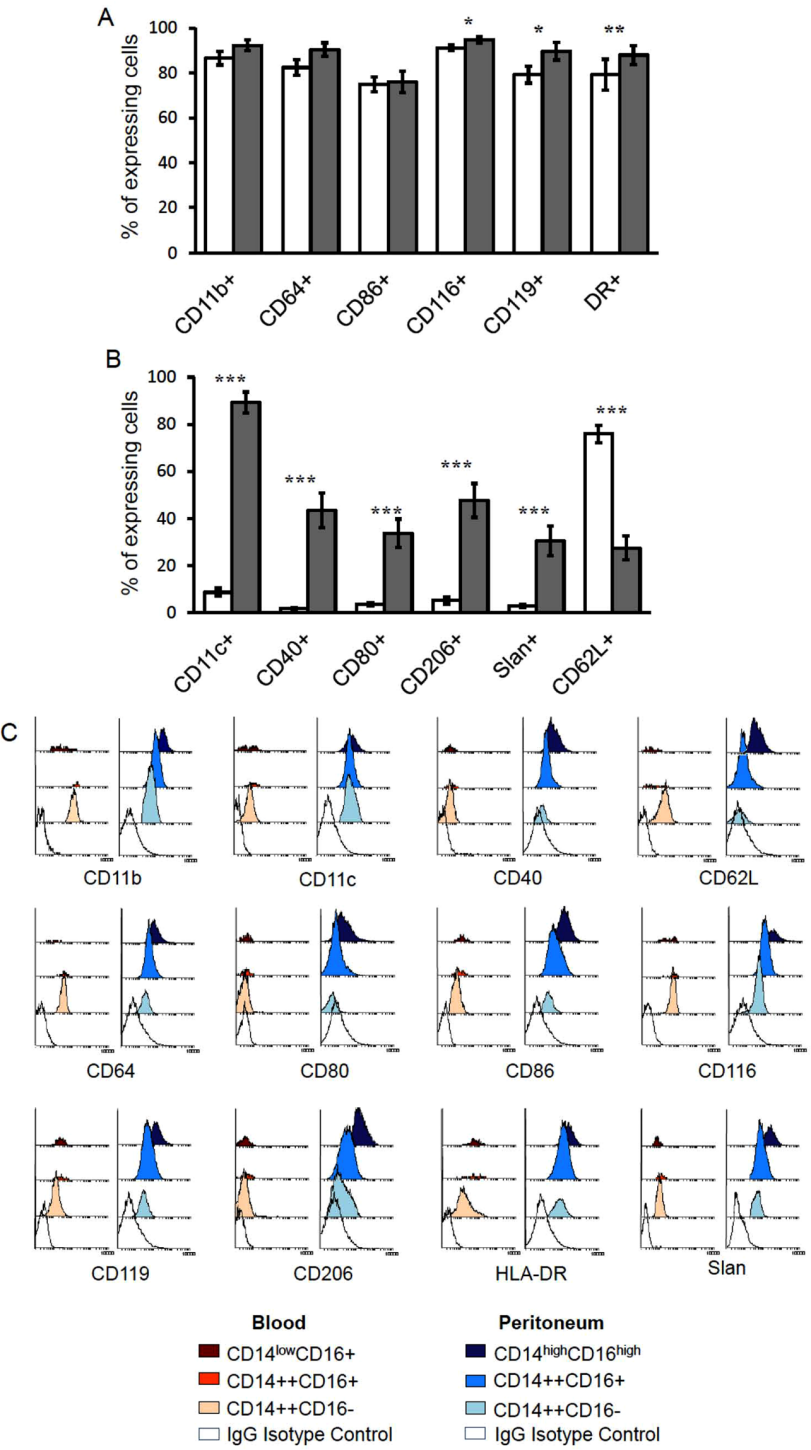
Flow Cytometry analysis of phenotypic characteristics of human peritoneal monocyte/macrophages compared to blood monocytes. Percentages of cells expressing several membrane markers were analyzed by FACS. A comparative analysis between blood monocytes and peritoneal monocyte/macrophages is displayed. Results on different frequencies of expression were grouped according to the similarity of expressing cells in blood and peritoneum (**A**), or to a high difference (**B**) between locations. Histograms represent the percentage of positive cells for every membrane marker indicated as mean ± SEM percentage of positive cells referred to the totality (100%) of monocyte/macrophage cells. White bars represent positive blood monocytes and grey bars positive peritoneal monocyte/macrophages. Mann-Whitney U test: *p < 0.05, **p < 0.01, ***p < 0.001, between locations. Representative FACS mean fluorescence intensity (MFI) histograms of blood monocytes and peritoneal monocyte/macrophages for every membrane marker analyzed in the different cells subpopulations are displayed (**C**).

To explore the functionality of pMo/Mφ, we analyzed by flow cytometry the expression of several surface markers on the whole population of pMo/Mφ comparatively to blood monocytes. Concretely, we analyzed receptors involved in activation: CD64 (FcγRI), CD40, CD80, CD86, CD116 (GM-CSFR) and CD119 (IFNγR1 or IFNγ chain α receptor); phagocytosis of complement-opsonised particles and adherence: CD11b and CD11c (the α subunit of CR3 and CR4 respectively); adhesion and migration: CD62L (Selectin-L) and P-selectin glycoprotein ligand-1(PSGL-1) carbohydrate modified Slan (6-sulfo LacNAc); antigen presentation: HLA-DR, and the M2 macrophage-polarized marker involved in phagocytosis: CD206 (mannose receptor). Results showed that some of them, such as CD11b, CD64, CD86, CD116, CD119 and HLA-DR were widely expressed in both peritoneal cells and blood monocytes with percentages ranging from 76.0 ± 4.7% for CD86 to 94.7 ± 1.3% for CD116 (Fig. 2A). Among them, CD116, CD119 and HLA-DR presented slightly higher, but significant, percentages in peritoneal cells. Others like CD11b and CD86, followed the same trend, although did not reach significant differences or were close to reach them, as it was the case of CD64 (p = 0.055) (Fig. 2A). Other markers presented remarkable differences between cells isolated from each location (Fig. 2B). The majority of those membrane molecules were significantly higher expressed in peritoneal cells than in blood monocytes, in which most of those markers could be actually considered absent. That was the case of CD11c, the co-stimulatory molecules CD40 and CD80, the mannose receptor CD206 and Slan (Fig. 2B). On the contrary, the percentage of cells expressing the adhesion molecule CD62L was significantly higher in blood monocytes (75.9 ± 3.7%) than in pMo/Mφ (27.4 ± 5.1%) (Fig. 2B).

A deeper analysis of the phenotypic profile of each CD14/CD16 subpopulation of pMo/Mφ and blood monocytes is summarized in Table 2. These data showed that the higher cell complexity and expression of CD16 in peritoneal cells goes parallel to the higher expression of all markers assayed, not only for those that already were higher in peritoneal cells than in blood monocytes (CD116, CD119 and HLA-DR), but also for those that did not show significant differences (CD11b, CD64 and CD86) (Fig. 2C). Even in the case of CD62L, that displayed percentages of CD62L+ cells higher in blood than in peritoneal cells (Fig. 2B), the percentage of CD62L+ peritoneal cells increased along with the cell complexity and expression of CD16 (Table 2). Nevertheless, in blood monocytes subsets the trend was variable depending on the marker analyzed. The majority of them showed a similar trend to peritoneal cells, increasing their frequencies as the expression of CD16 raised, except for CD11b, CD62L, CD64 and CD116, which showed the opposite trend, i.e., the higher percentage of positive cells for those markers appeared into the classic CD14^++^CD16^−^ subset. As displayed in Table 2 the percentages of positive cells for the analyzed markers were significantly different when comparing the corresponding peritoneum and blood cell subsets (*p < 0.05, **p < 0.01, ***p < 0.001) with some exceptions. Differences between subsets located in each compartment were significant for those molecules that were practically absent in blood monocytes, such as CD11c, CD40, CD80, CD206 and the exclusive and partial marker of the non-classical subset Slan, but also for CD62L, which was poorly expressed in peritoneal cells and enriched in blood monocytes. Nevertheless, differences between subsets in each location were also observed for widely expressed molecules, especially for those with an opposite trend of expression in blood and peritoneum, such as CD11b, CD64, CD116 and HLA-DR.

**Table 2:** Phenotypic profile of total pMo/Mφ and CD14/CD16 subsets compared to blood of healthy humans.

Markers	Total CD14+ cells		CD14/CD16 expression subsets					
	Blood	Peritoneum	Blood monocytes			pMo/Mφ		
			CD14^++^CD16^−^	CD14^++^CD16^+^	CD14^+/low^CD16^+^	CD14^++^CD16^−^	CD14^++^CD16^+^	CD14^high^CD16^high^
CD11b+	86.6 ± 3.1	**92.4 **±** 2.4**	87.6 ± 3.6	77.9 ± 4.2	51.5 ± 7.3	**77.9 **±** 5.1**	97.0 ± 1.3**	**99.2 ± 0.5**
CD11c+	8.6 ± 1.7	**89.2 **± 4.5***	6.6 ± 1.5	24.3 ± 4.7	27.1 ± 6.0	**77.2 **± 5.2***	91.5 ± 2.6***	**95.1 ± 3.0**
CD40+	1.8 ± 0.4	**43.4 **± 7.3***	1.8 ± 0.5	2.3 ± 0.6	4.0 ± 2.1	**21.2 **± 4.9***	60.7 ± 8.2***	**87.7 ± 6.2**
CD62L+	75.9 ± 3.7	**27.4 **± 5.1***	81.9 ± 4.1	48.8 ± 4.5	25.5 ± 5.4	**11.7 **± 2.6***	29.4 ± 5.7**	**66.4 ± 8.9**
CD64+	82.4 ± 3.5	**90.4 **±** 3.1**	94.4 ± 1.9	63.4 ± 3.5	19.2 ± 3.1	**84.7 **± 1.7***	97.2 ± 0.5***	**99.4 ± 0.2**
CD80+	3.4 ± 0.7	**33.7 **± 6.0***	7.0 ± 4.0	4.0 ± 1.0	4.8 ± 1.8	**9.8 **±** 3.4**	36.9 ± 7.5***	**78.6 ± 7.5**
CD86+	74.9 ± 3.8	**76.0 **±** 4.7**	71.9 ± 5.0	89.6 ± 2.4	91.0 ± 5.1	**56.2 **± 5.8*	95.9 ± 1.3*	**98.9 ± 0.6**
CD116+	91.2 ± 1.2	**94.7 **± 1.3*	95.7 ± 0.7	70.4 ± 4.6	41.2 ± 4.4	**89.1 **± 2.2**	**98.8** ± 0.4***	**99.8 ± 0.1**
CD119+	79.2 ± 3.7	**89.7 **± 4.0*	77.4 ± 3.9	90.8 ± 1.6	91.4 ± 1.3	**81.6 **±** 5.0**	97.9 ± 1.3*	**99.3 ± 0.5**
CD206+	5.2 ± 1.5	**47.6 **± 7.2***	4.7 ± 1.3	11.6 ± 3.0	11.0 ± 4.3	**28.2 **± 6.6***	60.3 ± 6.1***	**92.8 ± 1.4**
HLA-DR+	79.2 ± 6.9	**88.0 **± 4.2**	77.2 ± 7.4	92.8 ± 2.2	96.3 ± 0.8	**81.2 **± 4.8*	97.7 ± 1.1**	**97.5 ± 1.8**
Slan+	1.7 ± 0.5	**7.0 **± 1.0***	0.7 ± 0.4	3.4 ± 0.9	11.9 ± 2.7	**2.8 **± 0.6**	7.5 ± 1.1**	**12.8 ± 1.9**

Data represent the Mean ± SEM percentage of positive cells for every membrane marker analyzed. *p < 0.05, **p < 0.01, and ***p < 0.001 between percentages of positive pMo/Mφ CD14^++^CD16^−^ and CD14^++^CD16^+^ cells compared to their blood equivalent subsets.

We also analyzed the density of each receptor expressed per cell (MFI) in every cell subset in both blood and peritoneal cells (Table 3). A representative side by side experiment of markers expressed in cell subsets from both compartments is shown in Fig. 2C. Results confirm the gradual increased amount of molecules expressed on the membrane of pMo/Mφ as the complexity and expression of CD16 also increase. Hence, histograms corresponding to the more complex CD14^high^CD16^high^ peritoneal subpopulation (dark blue colour in Fig. 2C) are the most displaced to the right in all cases. The figure also remarks that percentages of cells expressing each marker analyzed are related to the density of receptors expressed per cell. Hence, those markers that showed a similar (CD11c, CD86, CD119 and HLA-DR) or opposite (CD11b, CD62L, CD64 and CD116) trend in the frequencies of expressing cells on each subset from both locations, followed a similar/opposite tendency for the intensity expressed per cell (MFI), respectively.

**Table 3:** Median fluorescence intensity of total pMo/Mφ and CD14/CD16 subsets compared to blood of healthy humans.

Markers	Total CD14+ cells		CD14/CD16 expression subsets					
	Blood	Peritoneum	Blood monocytes			pMo/Mφ		
			CD14^++^CD16^−^	CD14^++^CD16^+^	CD14^+/low^CD16^+^	CD14^++^CD16^−^	CD14^++^CD16^+^	CD14^high^CD16^high^
CD11b+	158.6 ± 31.2	**135.9 **±** 36.1**	133.9 ± 12.5	128.1 ± 17.6	59.7 ± 7.6	**67.2 ± 15.6** ^*^	**142.8 **±** 28.6**	**303.1 ± 49.4**
CD11c+	8.3 ± 1.4	**64.5 **± 8.9***	8.6 ± 2.2	22.9 ± 6.6	23.8 ± 8.6	67.8 ± 12.6***	**58.11 **± 8.5***	**95.5 ± 12.8**
CD40+	3.5 ± 0.5	**14.7 **± 4.1**	3.5 ± 0.9	4.3 ± 1.0	4.0 ± 1.0	**6.0 ± 1.5**	**18.2 **± 3.6***	**57.0 ± 12.2**
CD62L+	29.9 ± 3.7	**6.7 **± 1.4***	31.2 ± 3.7	15.9 ± 2.7	9.9 ± 1.7	3.1 ± 0.6***	**7.3 **± 1.4*	**22.5 ± 5.9**
CD64+	30.2 ± 2.6	**28.7 **±** 3.7**	31.7 ± 2.8	22.1 ± 2.0	5.7 ± 0.6	16.6 ± 1.8***	**30.6 **± 3.7*	**84.2 ± 12.5**
CD80+	2.5 ± 0.6	**7.0 **± 1.7**	2.7 ± 0.5	3.0 ± 0.6	3.2 ± 0.5	**2.8 ± 0.3**	**8.2 **± 1.6**	**22.1 ± 6.6**
CD86+	16.4 ± 1.6	**44.1 **± 11.0**	15.4 ± 1.4	23.9 ± 2.4	26.3 ± 2.3	**13.7 ± 1.5**	**57.7 **± 9.8***	**162.0 ± 27.1**
CD116+	45.6 ± 2.8	**67.8 **± 11.5*	47.2 ± 3.1	40.7 ± 4.7	19.8 ± 2.8	**44.5 ± 5.0**	**72.2 **± 9.9**	**161.3 ± 25.9**
CD119+	16.2 ± 2.3	**48.7 **± 8.11***	13.1 ± 2.7	21.7 ± 5.4	20.2 ± 2.4	**24.1 ± 2.3**	**62.2 **± 8.7**	**132.6 ± 29.6**
CD206+	2.9 ± 0.4	**23.0 **± 4.3***	2.9 ± 0.4	3.9 ± 0.5	4.4 ± 0.7	10.1 ± 1.1***	**25.1 **± 4.2***	**123.7 ± 22.5**
HLA-DR+	18.7 ± 3.9	**84.4 **± 16.0***	16.7 ± 3.4	60.0 ± 21.0	52.8 ± 9.6	55.6 ± 10.0***	**113.1 **±** 25.9**	**238.8 ± 51.6**
Slan+	8.4 ± 1.9	**44.8 **± 8.0***	8.4 ± 1.9	8.7 ± 1.9	6.6 ± 1.0	29.9 ± 0.1***	**30.8 **± 0.8***	**33.3 ± 1.4**

Data represent the Mean ± SEM of median fluorescence intensity (MFI) for each membrane marker analyzed. *p < 0.05, **p < 0.01, and ***p < 0.001, ratios of positive monocyte/macrophages from peritoneum compared to monocytes from blood of healthy subjects.

### Expression of GATA6 in human peritoneal macrophages

**Figure 3:**
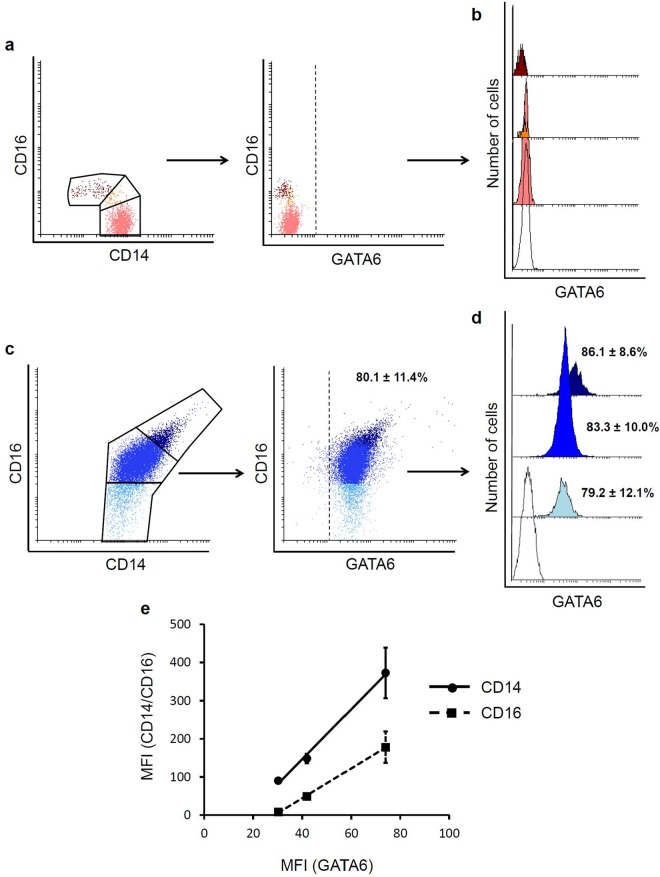
Flow cytometry analysis of the intracellular expression of transcription factor GATA6 in human peritoneal monocytes/macrophages. Representative FACS dot-plots of the gating strategy for blood monocytes subsets based on CD14/CD16 expression and the level of intracellular expression of transcription factor GATA6 in blood monocytes are displayed (**a**). Representative FACS histograms of intracellular expression of transcription factor GATA6 in the three blood monocytes subsets are shown partially overlaid (**b**). Representative FACS dot-plots of the gating strategy of peritoneal subsets based on CD14/CD16 expression, and the level of intracellular expression of transcription factor GATA6 in peritoneal monocytes/macrophages are also displayed (**c**). Representative FACS histograms partially overlaid corresponding to GATA6 intracellular expression in the three different peritoneal monocytes/macrophages subsets are shown (**d**). Percentages of GATA6 positive cells present in peritoneum, indicated as mean ± SEM, are shown next to the corresponding histogram. Percentages referred to the totality (100%) of monocyte/macrophages into the correspondent subset. Empty histograms correspond to FMO control-PE and coloured histograms to GATA6-PE in blood (pink: CD14^++^CD16^−^ classic subset; orange: CD14^++^CD16^+^ intermediate subset; dark red: CD14^+/low^CD16^+^ non-classical subset) or peritoneum (light blue: CD14^++^CD16^−^ subset; bright blue: CD14^++^CD16^+^ subset; dark blue: CD14^high^CD16^high^ subset). Correlation analysis of GATA6 intensity expression (MFI) related to the corresponding MFI of CD14 (black circles) and CD16 (black squares) for each monocyte/macrophage subset is displayed (**e**).

Trying to discriminate between mature resident peritoneal macrophages and newly migrated blood monocytes, we analyzed the intracellular expression of transcription factor GATA6, which has been described as a central regulator of the murine peritoneal macrophage phenotype. The results showed that while GATA6 was absent in peripheral blood monocytes (Fig. 3a,b), the percentage of GATA6+ cells in the peritoneal cavity was rather high (80.1 ± 11.4%) (Fig. 3c). Next, we analyzed the percentages of GATA6+ cells within each CD14/CD16 peritoneal subset. As displayed in Fig. 3d, the results did not show significant differences in the ratio of positive cells among the three peritoneal subpopulations; although there was a trend toward a higher number of GATA6+ cells within the more complex CD14^high^CD16^high^ subpopulation (86.1 ± 8.6%), followed by the intermediate subset (83.3 ± 10.0%), and the classic-like CD14^++^CD16^−^ subpopulation (79.2 ± 12.1%). Representative FACS histograms and quantitative analysis of GATA6 MFI within each pMo/pMφ subpopulation are displayed in Fig. 3d. Similarly to the percentages of GATA6 positive cells, the intensity level of cell expression within each peritoneal subset was directly related to the cell complexity, and therefore to the expression of CD14 and CD16. The analysis of GATA6 intensity expression (MFI) related to the corresponding MFI of CD14 and CD16 showed a clear correlation (R^2^ = 0.9957 and R^2^ = 0.9992, respectively) (Fig. 3e). Thus, taking into account the results reported from the mouse model, these data reveal that, in spite of their phenotypic differences, the majority of resident pMo/Mφ in homeostasis highly express the transcription factor GATA6. The mature and more active cells would mostly correspond to the complex CD14^high^CD16^high^ and, in a lesser extension, to the CD14^++^CD16^+^ intermediate subset; while the classic-like subset, which includes the higher percentage of GATA6^−^ small peritoneal cells would be likely recruited from blood monocytes that have just migrated into the peritoneal cavity.

### Phagocytic activity of human peritoneal CD14/CD16 subpopulations

**Figure 4:**
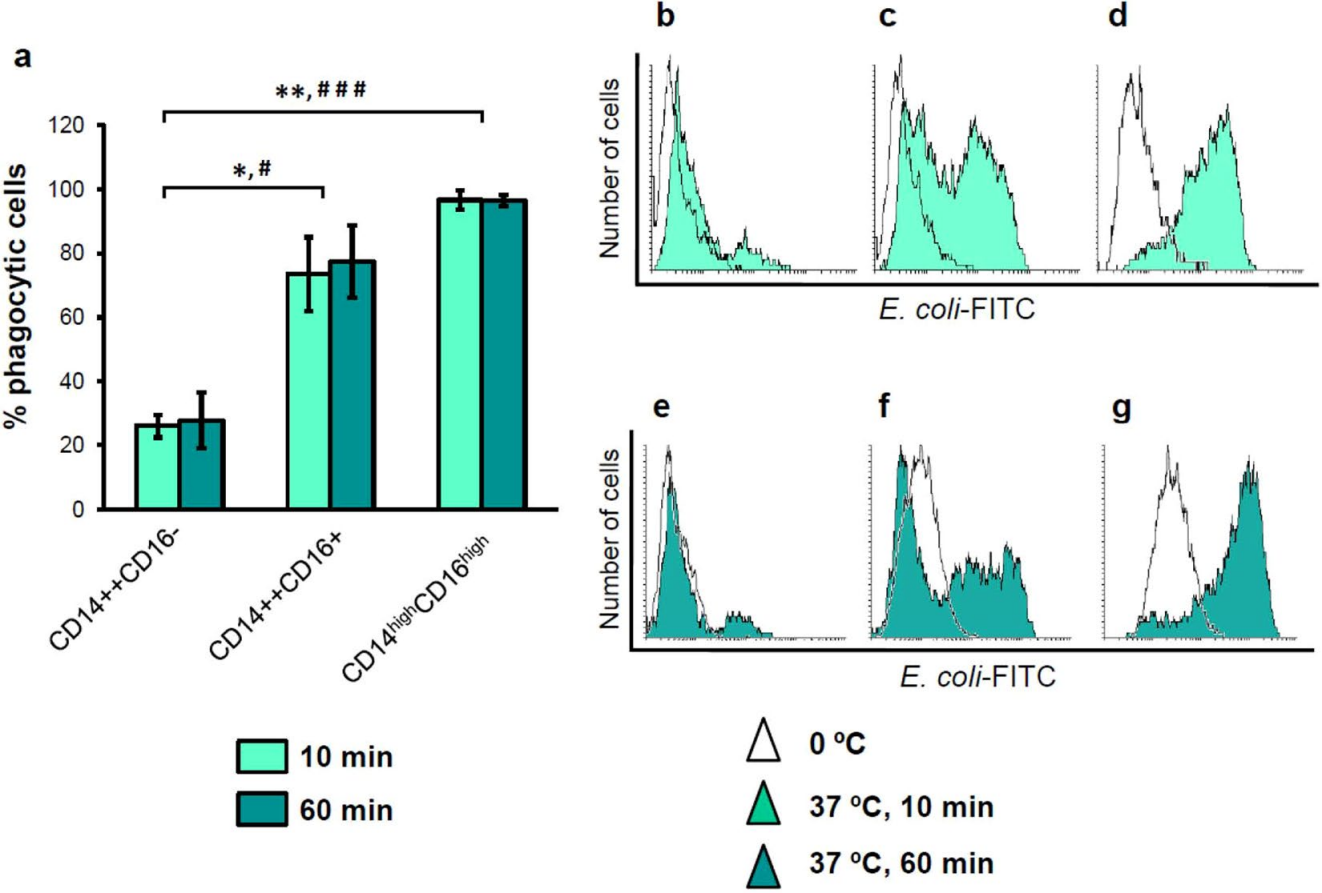
Flow cytometry analysis of the phagocytic activity of human peritoneal monocyte/macrophages. Phagocytosis was analyzed by measuring intracellular FITC-labelled *E. coli* fluorescence. Percentages of phagocytic cells were comparatively analyzed among the three different peritoneal subpopulations after incubation with the FITC-labelled *E. coli* for 10 minutes (bright aquamarine bars) and 1 hour (dark aquamarine bars) (**a**). Bars represent percentages of phagocytic cells into the three peritoneal subsets indicated as mean ± SEM percentage of FITC-positive cells referred to the totality (100%) of cells into the correspondent subset. Mann-Whitney U test among the three subsets: *p < 0.05, **p < 0.01, for the percentage of phagocytic cells after 10 min incubation; and ^#^p < 0.05, ^###^p < 0.001, for the percentage of phagocytic cells after 1 hour incubation with FITC-labelled *E. coli*. Representative FACS histograms of engulfed bacteria per cell, measured as fluorescence in FITC channel, for every peritoneal subset are also displayed for 10 min assays: CD14^++^CD16^−^ (**b**), CD14^++^CD16^+^ (**c**) and CD14^high^CD16^high^ (**d**); and for 1 hour assays: CD14^++^CD16^−^ (**e**), CD14^++^CD16^+^ (**f**) and CD14^high^CD16^high^ (**g**). In all cases empty histograms correspond to control-FITC signal obtained at 0 °C and coloured histograms to FITC signal at 37 °C for the corresponding time points.

Flow cytometry analysis of phagocytic activity of pMo/Mφ subpopulations showed that the percentage of phagocytic cells in the classical-like subset was 26.0 ± 3.7% after 10 min incubation with *E. coli* at 37 °C, and 27.9 ± 8.7% after 1 hour at 37 °C. Subsets of CD16^+^ cells engulfed significantly more bacteria than CD16^−^ cells did. Thus, the intermediate subset showed percentages of phagocytic cells of 73.5 ± 11.5% and 77.4 ± 11.2% for 10 min and 1 hour assays, respectively; while the more complex subset displayed the highest frequency of phagocytic cells (96.6 ± 3.0%) even after only 10 min assays, which was maintained (96.4 ± 1.8%) after 1 hour incubations (Fig. 4a). Concomitantly to the percentages of phagocytic cells, intracellular bacterial load, measured as MFI, was higher as it was the complexity and the size of the cell subsets. Thus, as it can be observed in the representative histograms displayed in Fig. 4b,e the classic-like subset showed the lowest MFI, both after 10 min (bright aquamarine histogram in Fig. 4b) and 1 hour assays (dark aquamarine histogram in Fig. 4e), the intermediate subset showed an intermediate MFI (Fig. 4c,f), and the complex subset presented the highest MFI levels (Fig. 4d,g).

### Oxidative potential of pMo/Mφ

**Figure 5:**
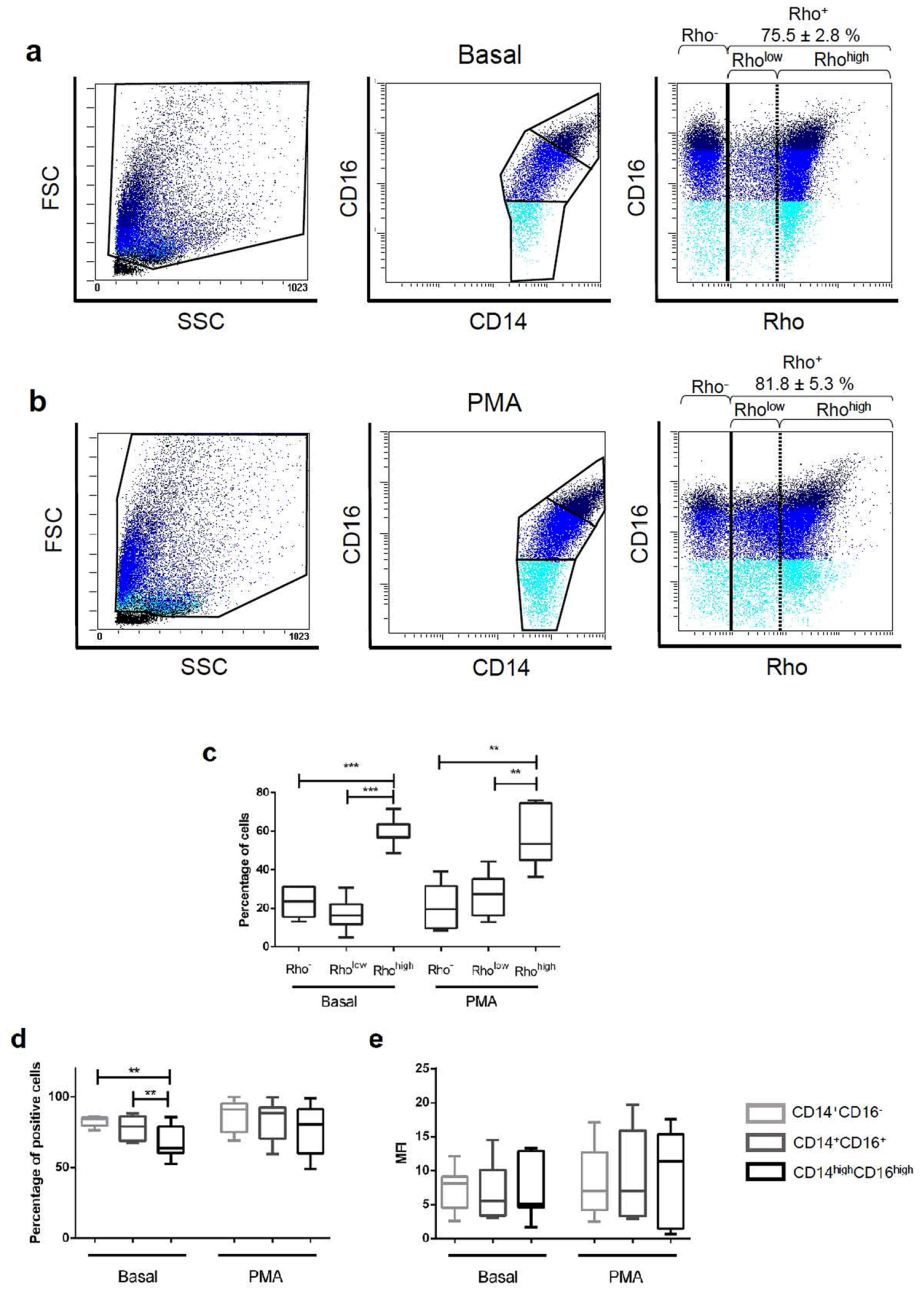
Flow cytometry analysis of the oxidative potential of human peritoneal monocyte/macrophages. Oxidative potential was measured by using a dihydrorhodamine 123 (DHR)-based assay. Positive oxidative cells were detected following ROS oxidation of DHR to green fluorescent rhodamine 123 (Rho). Representative FACS dot-plots of unstimulated (basal) peritoneal cells incubated with DHR are displayed. Dot-plots panels show forward/side scatter distribution of cells, CD14/CD16 gating, and Rho production in each cell subset (**a**). The same representative information is represented for PMA-stimulated cells (**b**). Light blue: CD14^++^CD16^−^ subset; bright blue: CD14^++^CD16^+^ subset; dark blue: CD14^high^CD16^high^ subset. The black line corresponds to the division between positive and negative cells (percentages of Rho-positive cells are displayed); the dotted line indicates the two different Rho^+^ subsets in terms of expression: Rho^low^ and Rho^high^. Boxes and whiskers graphic represents: Percentages of cells subsets separated by Rho expression for basal and PMA-stimulated cells. Mann-Whitney U test among the three Rho subsets: **p < 0.01, ***p < 0.001 (**c**); Distribution (%) of Rho-positive cells into the three CD14/CD16 subsets, for basal and PMA-stimulated cells. Mann-Whitney U test among the three CD14/CD16 subsets: **p < 0.01 (**d**); Rho MFI of the three CD14/CD16 subsets (**e**).

Oxidative capacity of pMo/Mφ was also measured by flow cytometry. To this purpose, dihydrorhodamine 123 (DHR) was added to cell samples, which displays green fluorescence when is oxidized to rhodamine 123 (Rho) by the ROS generated by cells, indicating their oxidative potential. Two parallel assays were performed with each sample, one without stimulation, to test the basal state of pMo/Mφ regarding its oxidative activity, and another one with cells stimulated with PMA. In the first case a 75.5 ± 2.8% of cells showed oxidative potential (Fig. 5a), and no significant differences were found with those stimulated with PMA (81.8 ± 5.3%) (Fig. 5b).

Surprisingly, when pMo/Mφ were classified in terms of rhodamine oxidative capacity three different subpopulations were observed, i.e., a population with no oxidative capacity: Rho^−^, and two subpopulations with different level of oxidation: Rho^low^, and Rho^high^. These three subpopulations were detected both in basal conditions (Fig. 5a) and after PMA stimulation (Fig. 5b). Percentages of Rho^high^ cells were significantly higher in both basal and PMA-activated conditions, while the frequency of Rho^−^ cells was low and similar in both cases. However, while percentages of Rho^low^ showed a trend to be slightly lower than the corresponding to Rho^−^ cells in basal conditions, this tendency was opposite in PMA-treated cells (Fig. 5c).

When cellular baseline oxidative potential was separately analyzed within each established CD14/CD16 subset, we found that the complex CD14^high^CD16^high^ cells showed a lower percentage of oxidative cells (66.9 ± 4.4%) than both the classic-like CD14^++^CD16^−^ (82.7 ± 1.1%, p = 0.0251) and the intermediate CD14^++^CD16^+^ (78.3 ± 3.4%, p = 0.0274) subsets. Contrarily, when peritoneal cells were stimulated with PMA differences between the percentages of Rho positive cells within each CD14/CD16 subset were not significant, revealing a similar distribution of oxidative cells under this condition (Fig. 5d). Nonetheless, in terms of MFI values, differences among the three subpopulations of pMo/Mφ were not significant (Fig. 5e).

### Analysis of intracellular expression of TNF-α, IL-6 and IL-10 cytokines in human peritoneal CD14/CD16 subpopulations

**Figure 6:**
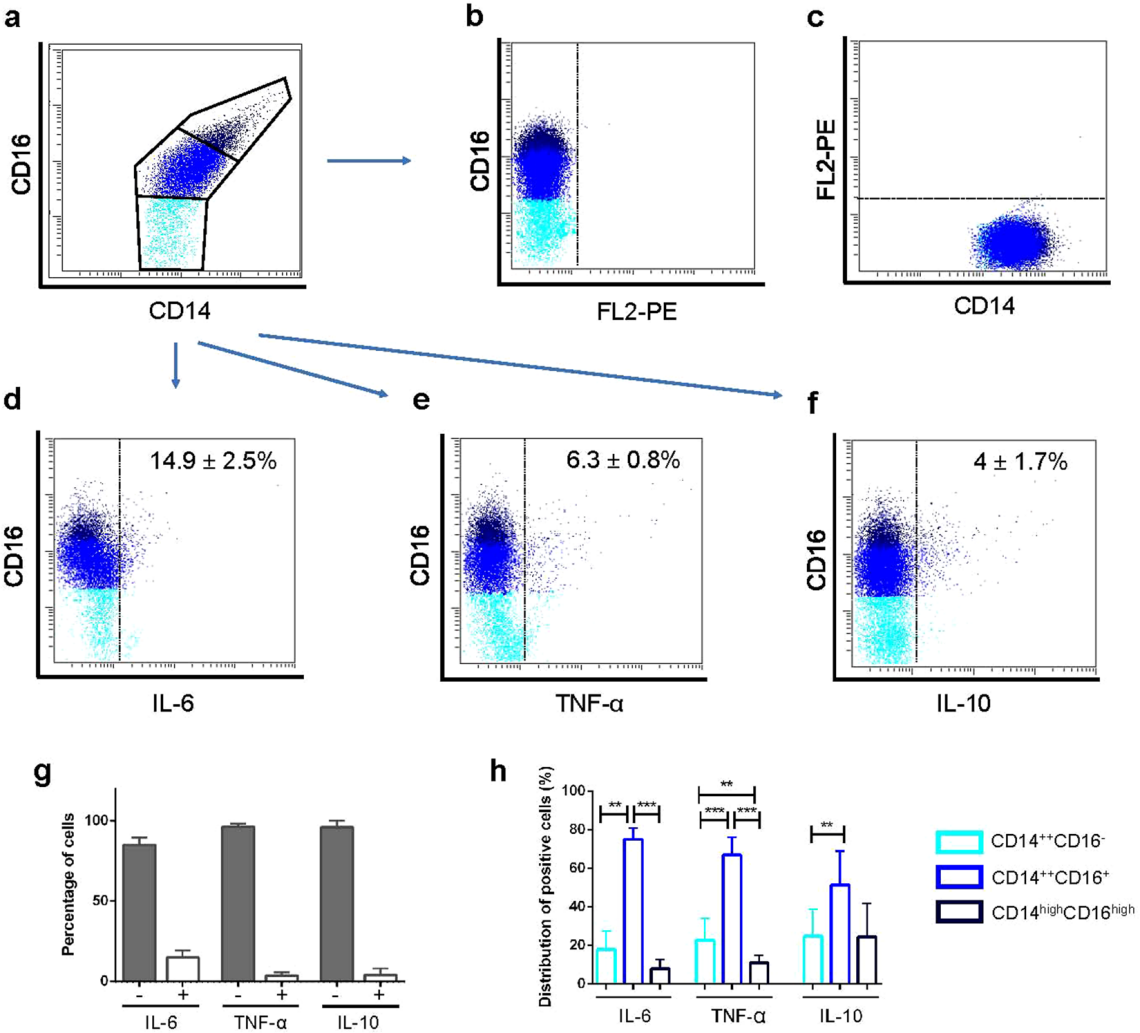
Flow cytometry determination of IL-6, TNF-α and IL-10 intracellular expression. Representative FACS dot-plots showing CD14/CD16 gating (**a**), Fluorescence Minus One (FMO) controls of PE channel (FL2) in which cells were stained with CD16-PE-Cy5 (**b**) and CD14-FITC (**c**), and intracellular expression of cytokines IL-6 (**d**), TNF-α (**e**) and IL-10 (**f**) related to CD16 cell membrane expression are displayed. Bars showing percentages of cytokine-negative (grey bars) and cytokine-positive (white bars) peritoneal monocyte/macrophages are displayed as mean ± SEM (**g**). Empty bars with coloured edges represent distribution of cytokine-positive cells into the three different CD14/CD16 subsets as mean ± SEM (%) (**h**). Mann-Whitney U test for the percentages of cytokine-positive cells among the three CD14/CD16 subsets: **p < 0.01, ***p < 0.001 (**e**). Light blue: CD14^++^CD16^−^ subset; bright blue: CD14^++^CD16^+^ subset; dark blue: CD14^high^CD16^high^ subset.

Results from the analysis of the intracellular content of cytokines in the three CD14/CD16 subsets of pMo/Mφ (Fig. 6a) compared to the corresponding intracellular staining FMOs (Fig. 6b,c) (Gating strategy displayed in Supplementary Fig. S1), showed that the expression of IL-6 (Fig. 6d), TNF-α (Fig. 6e) and IL-10 (Fig. 6f) was very low in these peritoneal cells, finding 14.9 ± 2.5% of IL-6+ cells, 6.3 ± 0.8% of TNF-α+ cells and 4 ± 1.7% of IL-10+ cells (Fig. 6g).

When percentages of positive cells for these cytokines were analysed and compared among each CD14/CD16 subpopulation (Fig. 6h), we found that the highest ratios of cytokine-positive cells corresponded to the intermediate CD14^++^CD16^+^ subset for the three cytokines. Furthermore, differences between the CD14^++^CD16^+^ subset compared with their counterparts were significant for the pro-inflammatory cytokines IL-6 and TNF-α; although for the anti-inflammatory IL-10 this difference was only significant compared with percentages of IL-10+ CD14^++^CD16^−^ cells. In turn, differences between percentages of cytokine-positive cells within the classic-like CD14^++^CD16^−^ compared with those obtained within the complex CD14^high^CD16^high^ subsets were only significant for TNF-α.

## Discussion

Although tissue-resident macrophages are known for more than 100 years, the studies on the physiology of this cell type in humans have been hindered by the difficulty of isolating macrophages in conditions of health and disease from peripheral tissues, which endures aggressive surgical interventions in which besides that few cells are obtained they are difficult to grow *in vitro*. For this reason, the majority of research on resident macrophages in both steady-state and disease has been carried out in experimental animals, especially in the mouse model. However, extrapolation from mice to man is not always feasible, which is especially true in this particular subject^21,22^. Furthermore, studies on the role of the immune system in inflammatory diseases in humans largely rely on findings obtained from the blood compartment. However, studying macrophages from an inflammatory scenario can make valuable contributions to a better understanding of the physiopathology of those human diseases^2,19^. Hence, it seems necessary to assess whether the local tissue mediators and macrophage transcription factors that have been identified in mice play equal roles in the biology and development of humans tissue-resident macrophages. Human pMo/Mφ are a good source of this cell type to study their biological properties under steady-state conditions. These samples must be obtained from healthy people or patients whose disease does not directly involve the peritoneal cavity. The most frequent examples are women subjected to gynaecological laparoscopy/laparotomy or continuous ambulatory peritoneal dialysis (CAPD) patients not affected by spontaneous bacterial peritonitis (SBP), a complication that occurs frequently in patients with cirrhosis and ascites and less frequently in CAPD^23–26^. Nevertheless, the required surgical procedures to obtain peritoneal samples from healthy people usually yield very low cell numbers, so that performing functional studies based on cell cultures remains as a challenging task. For this reason, there is little information about the phenotype and function of pMo/Mφ from normal individuals. In our aim to characterize normal human pMφ according to level 1 (developmental characteristics) and level 2 (surface antigen expression, phagocytosis and oxidative activity and intracellular cytokines content) proposed by Guilliams *et al*.^27^ to define a new myeloid cell type, we have compared several features of pMo/Mφ with the well defined CD14/CD16 blood monocytes subsets to describe common features or tissue-specific characteristics and differences. We found pMo/Mφ to represent a heterogeneous cell type based on both morphology and expression of CD14/CD16. It is integrated by similar proportions (approx. 42%) of small classical-like (CD14^++^CD16^−^) and intermediate-like (CD14^++^CD16^+^) cells, and a new subset of highly complex CD14^high^CD16^high^ cells (approx. 16%), which is absent in peripheral blood. In turn, cells similar to non-classical blood monocytes are not found into the peritoneal cavity. In regards to the staining pattern for both CD14 and CD16, pMo/Mφ exhibit a higher expression level than blood monocytes, which would make them more efficient, or better equipped, to detect LPS and phagocyte microorganisms. Of note, the proportions of these subsets result modulated under inflammatory conditions, thus we have previously found in ascites of decompensated cirrhotic patients that percentages of CD14^high^CD16^high^ cells were increased up to 33 ± 2.4%, the CD14^++^CD16^+^ intermediate subset reached up to 49 ± 2.0%, while the CD14^++^CD16^−^ classical subset, went down to 18 ± 1.3%^17^, these modifications in pathology *vs*. steady-state conditions strength the relevance of the results presented here.

Furthermore, we tested the expression of various monocyte/macrophage-associated surface antigens involved in phagocytosis of IgG-opsonised (CD64, high affinity FcγRI) and complement-opsonised particles (CD11b and CD11c, the α chains of Complement receptors, CR3 and CR4); adherence to stimulated endothelium and migration (CR3, CR4, CD62L and Slan), antigen presentation (MHC class II molecule HLA-DR), co-stimulatory molecules (CD80, CD86, CD40), cytokines receptors (CD116, GM-CSFR and CD119 IFNγR1 or IFNγ chain α receptor) and the mannose receptor (CD206) described as a M2 macrophage polarized marker and indicative of activation/maturation^28^. Compared with the whole population of blood monocytes, the expression of CD11b, CD64, and CD86 on pMo/Mφ was not statistically different, while we found small but significant differences for a higher expression of CD116, CD119 and HLA-DR on pMo/Mφ. The most striking differences were observed for CD11c, CD40, CD80, CD206, Slan and CD62L, with the exception that the last one was the only marker showing higher expression on blood monocytes. In view of these phenotypic results, one might assume that human pMo/Mφ would be able to exhibit a high antimicrobial (also supported by the phagocytic and oxidative capacity displayed by these cells), antigen–presenting and T-cell co-stimulatory capacity, although this remains to be further studied. On the other hand, the gradual increment of percentages of expression and density per cell of CD206 observed from 28.2% in CD14^++^CD16^−^ to 60.3% in CD14^++^CD16^+^ and 92.8% in CD14^high^CD16^high^ suggests that human resident pMo/Mφ may also display phenotypic and functional properties of M2 polarized macrophages previously reported from CAPD patients^29,30^ and endometriosis^31^. In this regard, a recent work carried out on experimental endometriosis in a mouse model has demonstrated the dynamic changes in the proportions and polarization profile (M1 and M2) of F4/80^hi^CD11b^hi^ large and F4/80^low^CD11^blow^ low pMφ subsets along with the development of the endometriosis lesions^32^. Nevertheless, the most striking differences between blood and peritoneal monocytes/macrophages subsets were found on percentages and MFI of cells expressing the selectin CD62L, i.e., blood monocytes decrease CD62L expression as CD16 increases, while percentages of pMφ expressing CD62L in each subset increase along with the expression of CD16. Furthermore, results obtained for the expression of Slan (6-sulfo LacNAc), reported as a marker differentiating new subsets of CD16^+^ monocytes that are expanded in patients with sarcoidosis (Slan-negative, CD16^+^) or depleted in hereditary diffuse leukodystrophy (Slan-positive, CD16^+^)^33^, showed that although the tendency is similar in both blood and peritoneal subsets, the overall percentages of Slan expressing cells were statistically higher in the latter compartment. Differences on expression of these adhesion molecules could be related with a very different pattern of cell-migration on each compartment (endothelium/mesothelium). In this regard, it has been recently described that GATA6+, F4/80+ mature pMφ from mice, rapidly infiltrate the injured liver through a non-vascular route, adopting an alternatively M2 activated phenotype and protecting against acute liver damage^34^.

Expression of GATA6 is absent in blood monocytes, while the percentage of GATA6 expressing cells among the three described subpopulations of pMo/Mφ is similar. Nevertheless there is a strong correlation between the gradual increase of GATA6 intracellular expression and the cellular membrane expression of both CD14 (R^2^ = 0.9957) and CD16 (R^2^ = 0.9992). These findings could either suggest that the migration of monocytes to the peritoneal cavity under steady-state conditions is very low, or that the expression of GATA6 in newly arrived peritoneal monocytes is very rapid. Altogether, these data and those reported from mice, point out to the more complex population of CD14^high^CD16^high^ pMo/Mφ as the phenotypic signature of mature differentiated human resident pMφ, while the intermediate subset CD14^++^CD16^+^ could represent a mixed transitional cell type also integrated by recruited blood monocytes developing phenotypic and functional characteristics of resident peritoneal macrophages. However, taken into account the results of Bain *et al*.^20^, on the origin, self-renewal and age-dependent replacement of F4/80^hi^CD11b^hi^ large pMφ by the F4/80^low^MHC-II^hi^ subset of small pMφ derived from inflammatory monocytes^20^, and without the possibility to perform fate-mapping experiments in humans, we can neither assume nor exclude the embryonic-derived origin of the CD14^high^CD16^high^ subset.

The low frequency of cells expressing intracellular IL-6, TNF-α and IL-10 cytokines confirms the homeostatic state of this cell population. Interestingly, the intermediate subset displays the highest proportions of intracellular cytokines, while the CD14^high^CD16^high^ subset presents a higher frequency of IL-10 positive cells compared to those of pro-inflammatory cytokines, which favours the hypothesis of its M2 polarization trend.

Finally, the linear relationship between the expression of CD14/CD16 and activation/maturation markers, such as CD206 and HLA-DR, the intracellular content of GATA6, the phagocytic/oxidative activity, and the intracellular content of IL-6, TNF-α and IL-10, support that the CD14^high^CD16^high^ subset is the mature phenotype of human resident pMφ in steady-state conditions, which would play a main role in the maintenance and recovery of homeostasis after injuries and pathogen challenges. The present results on healthy human pMo/Mφ provide a useful information for those researchers studying peritoneal macrophages under inflammatory conditions or in the presence of tumors.

## Material and Methods

### Study subjects and cell collection

Steady-state peritoneal cells of 79 healthy women were obtained from the Gynecological Unit of the Hospital Clínico Universitario Virgen de la Arrixaca, Murcia, Spain. Cell samples (from blood and peritoneal cavity) were obtained during exploratory or therapeutic laparoscopies for benign gynaecological pathology (simple ovarian cysts or uterine fibroids) or tubal ligation. Both during surgery and in the analysis of peritoneal lavage cells there was no evidence of involvement of the peritoneal space in any one of the patients. After opening the abdomen by incision, the peritoneal cavity was instilled with 50 mL phosphate-buffered saline (PBS) solution that was collected from the rectouterine pouch, or pouch of Douglas, strictly avoiding contamination by blood. Nevertheless red-coloured samples indicative of peripheral blood contamination were excluded. Abdominal surgery continued after this brief lavage procedure. Samples were then maintained at 4 °C to avoid cell attachment to plastic. Cells were finally washed with RPMI-1640 (GIBCO Invitrogen, Paisley, UK) and processed for flow cytometry analysis.

The ethics committees (Hospital Clínico Universitario Virgen de la Arrixaca, and Comité de Bioética de la Universidad de Murcia) approved the study protocol according to the 1975 Declaration of Helsinki and all peritoneal cells donors gave informed written consent to be included in this study.

### Flow cytometry analysis

Cells from human peritoneum were stained with monoclonal antibodies and analyzed by flow cytometry. Antibodies used were monoclonal mouse anti-human CD14-FITC and CD14-PE clone 61D3 (eBioscience, San Diego, CA, USA), CD3-FITC clone UCHT1, CD11b-PE clone 1CRF44(44), CD11c-FITC clone 3.9, CD16-PE-Cy5 clone 3G8, CD19-PE-Cy5 clone SJ25C1, CD33-PE clone WM53, CD40-FITC clone 5C3, CD62L-PE clone DREG-56, CD64-PE clone 10.1, CD80-FITCclone L307.4, CD86-PE clone 2331(FUN-1), CD116-PE clone hGMCSFR-M1, CD119-PE clone GIR-94, CD206-FITC clone 19.2, HLA-DR-FITC clone TÜ36 and Slan-PE clone DD-1 (BD-Pharmingen, San Diego, CA, USA). The mouse IgG1-FITC, IgG1-PE, IgG1-PE-Cy5 clone MOPC-21 and IgG2a-PE clone MOPC-173 antibodies used as isotype controls were from BD-Pharmingen. In brief, peritoneal cells were washed once with PBS and twice with RPMI-1640, before being resuspended at a ratio of 0.2·10^6^ total white cells in a volume of 100 μl RPMI-1640 and then stained with 5 μl of the corresponding monoclonal antibodies and incubated in the dark on ice, fixed with a fixing-lysing solution (Becton Dickinson, San José, CA, USA) and then washed twice with PBS. Prior to preparation for flow cytometry analyses, the viability of the resuspended samples was analyzed by optical microscopy, using Trypan Blue dyeing. Absence of cells aggregates after resuspension was also determined by microscopy observation. Finally, fixed marked cells were resuspended in PBS and kept at 4 °C in the dark until data acquisition. In the case of peripheral blood, 50 μl samples were directly used, with no prior treatment, and then stained as previously explained.

Alternatively, after following the same washing and preparation procedure, 0.4·10^6^ white cells were stained intracellularly with the IntraStain Kit (Dako, Glostrup, Denmark) following manufacturer instructions. Antibodies used for intracellular stain were rabbit monoclonal anti-human GATA6-PE clone D61E4 (Cell Signaling Technology, Danvers, MA, USA), mouse monoclonal anti-human IL-6-PE, TNF-α-PE (ImmunoTools, Friesoythe, Germany), and rat monoclonal anti-human IL-10-PE (BioLegend, San Diego, CA, USA).

Flow cytometry analyses were performed on three-color fluorescence FACS Canto II (Becton Dickinson, San José, CA, USA), Epics XL or FC500 (Beckman Coulter, Brea, CA, USA) using FACS Canto Software and Cytomics RXP Analysis Software respectively, or version 2.5.1 of Flowing Software. 30,000–200,000 gated events were acquired and analyzed. Leukocytes were gated based on FCS *vs*. SSC (Forward *vs*. Side Scatter) on a lineal scale, thus excluding death cells. Then, mononuclear myeloid cells were gated on the base of CD14 expression and morphology. This gate was used to analyze the rest of markers studied as described previously^17^. Subpopulations were measured as a percentage of total number of CD14^+^ cells and as a percentage of the total number of CD16^+^ cells. Cell aggregates were excluded based on FSC-W *vs*. FSC-A on a lineal scale. Besides isotype controls, FMO (Fluorescence Minus One) control assays were carried out in order to establish fluorescence control limits that ensure no spread of fluorescence between channels (Supplementary Fig. S1).

### Phagocytic activity analysis

Peritoneal cell samples were managed as explained above, by washing them once with PBS, twice more with RPMI-1640, and finally resuspended in RPMI-1640 at a ratio of 0.4·10^6^ total white cells in a volume of 100 μl. Phagocytic activity of pMo/Mφ was then tested with the Phagotest™ kit (BD-Pharmingen, San Diego, CA, USA), which contains fluorescein (FITC)-labelled opsonised *E. coli* bacteria, following manufacturer’s instructions, allowing phagocytosis at 37 °C for a short (10 min) and long time assay (1 hour). Cells were also stained with the correspondent monoclonal mouse anti-human antibodies for the characterization of pMo/Mφ subpopulations’ phagocytic activity. Flow cytometry analyses were then performed, as explained above, on three-color fluorescence FACS Canto II, and Epics XL or FC500 (Beckman Coulter) using Cytomics RXP Analysis Software or version 2.5.1 of Flowing Software. 30,000–200,000 gated events were acquired and analyzed. Percentage of positive phagocytic cells (positive for FITC channel) and MFI of the pMo/Mφ subpopulations were analyzed.

### Oxidative potential analysis

Oxidative potential of pMo/Mφ was measured by using a dihydrorhodamine 123 (DHR)-based assay. Peritoneal samples were washed once with PBS and then with DMEM. Cells were resuspended in DMEM at a ratio of 0.4·10^6^ total white cells in a volume of 50 μl. For each sample, two tests were performed in parallel, one with the addition of phorbol myristate acetate (PMA; Sigma Chemical Co., St Louis, MO) and another without PMA. Anti-CD14-PE and anti-CD16 antibodies were added to the sample, together with 2.5 μl of DHR 2.5 μg/mL (Merck, Darmstadt, Germany) and 10 μl of PMA 2 mg/mL. Samples were incubated for 15 minutes at 37 °C. Then, oxidative reaction was interrupted by placing the sample tubes on ice and cells were fixed with 1 mL of fixing-lysing solution. Cells were washed twice with PBS and kept at 4 °C in the dark until data adquisition. Flow cytometry analyses were then performed, as explained above. The produced ROS would oxidize DHR to rhodamine 123, which possess green fluorescence; thus, cells with oxidative potential exhibited green fluorescence.

### Statistical analysis

Results are reported as histograms representing mean ± standard error of the mean (SEM). The observations considered as outliers are not represented. Statistic differences were analyzed using Mann-Whitney U test and Wilcoxon signed-rank test. All reported p values are two-sided, and p values lower than 0.05 were considered to indicate statistical significance. All calculations were performed using the SPSS 19.0 and GraphPad Prism 6 software (Chicago, IL, USA).

Reporting of the study conforms to STROBE and EQUATOR guidelines^35^.

## Electronic supplementary material

Supplementary Figure S1.

## Data Availability

All data generated or analysed during this study are included in this published article (and its Supplementary Information files).
