# Supplementary material for: Characterization of human peritoneal monocyte/macrophage subsets in homeostasis: Phenotype, GATA6, phagocytic/oxidative activities and cytokines expression
Source: Sci Rep. 2018 Aug 24;8:12794. doi: 10.1038/s41598-018-30787-x (PMC6109155; doi:10.1038/s41598-018-30787-x)
Supplement: Supplementary file 1 — Supplementary Figure S1. [file 41598_2018_30787_MOESM1_ESM.pdf]

Supplementary Information

Characterization of human peritoneal monocyte/macrophage subsets in homeostasis: Phenotype, GATA6, phagocytic/oxidative activities and cytokines expression.

Antonio José Ruiz-Alcaraz[1,#], Violeta Carmona-Martínez[1,#], María Tristán-Manzano[1], Francisco Machado-Linde[2], María Luisa Sánchez-Ferrer[2], Pilar García-Peñarrubia[1,*,#] and María Martínez-Esparza[1,*,#]

[1] Departamento de Bioquímica, Biología Molecular (B) e Inmunología. Facultad de Medicina, IMIB and Regional Campus of International Excellence "Campus Mare Nostrum". Universidad de Murcia, Murcia, Spain.[2] Servicio de Ginecología y Obstetricia, Hospital Clínico Universitario Virgen de la Arrixaca, IMIB, Murcia, Spain.

[#] Equal contribution

[*] **Correspondence:**
Pilar García Peñarrubia
Phone: +34 868 884673
e-mail: pigarcia@um.es
María Martínez-Esparza
Phone: +34 868 883989
e-mail: maria@um.es
Fax: +34 868 888601

Supplementary Figure S1

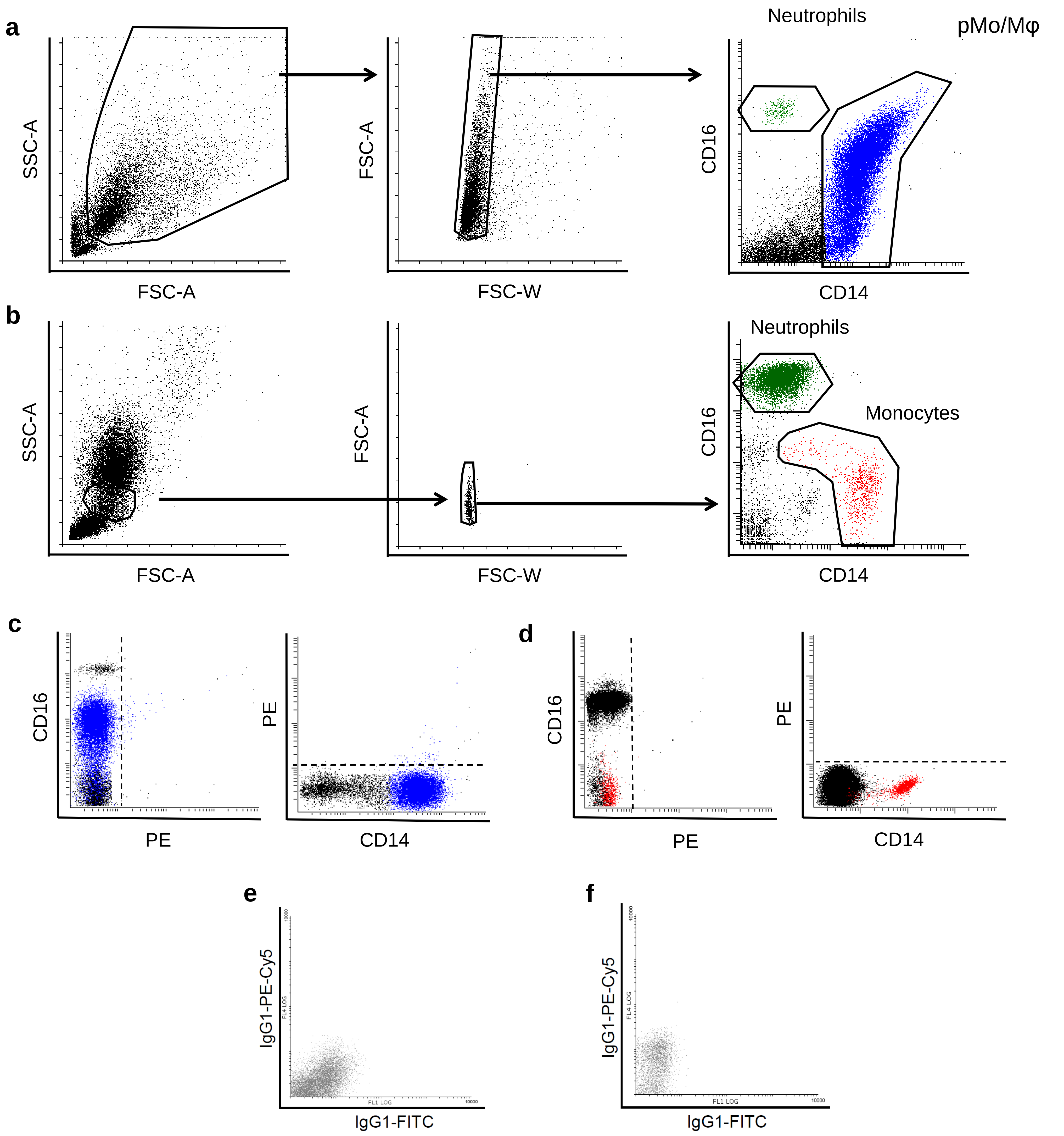

Supplementary Figure S1. Gating strategy of peritoneal cells: First, cells were selected according to morphology (forward vs. side scatter). Afterwards, cells aggregates were excluded based on FSC-W vs. FSC-A. Finally, cells were gated based on CD14/CD16 expression **(a)**. Same gating strategy was applied to blood cells **(b)**. Blue events represent pMo/pMφ, red ones represent blood monocytes and green events represent neutrophils. FMO control assays were carried out in order to establish fluorescence control limits that ensure no spread of fluorescence between channels. Representative FACS dot-plots of Fluorescent Minus One (FMO) of PE channel, in which cells were stained with CD14-FITC and CD16-PE-Cy5, are displayed for both peritoneal **(c)** and blood cells **(d)**. Representative dot-plots of isotype controls of peritoneal **(e)** and blood cells **(f)** are also displayed.
